# Community interactions and spatial structure shape selection on antibiotic resistant lineages

**DOI:** 10.1371/journal.pcbi.1006179

**Published:** 2018-06-21

**Authors:** Sylvie Estrela, Sam P. Brown

**Affiliations:** 1 Department of Ecology and Evolutionary Biology, Yale University, New Haven, Connecticut, United States of America; 2 Microbial Sciences Institute, Yale University, West Haven, Connecticut, United States of America; 3 School of Biological Sciences, Georgia Institute of Technology, Atlanta, Georgia, United States of America; CNRS, FRANCE

## Abstract

Polymicrobial interactions play an important role in shaping the outcome of antibiotic treatment, yet how multispecies communities respond to antibiotic assault is still little understood. Here we use an individual-based simulation model of microbial biofilms to investigate how competitive and mutualistic interactions between an antibiotic-resistant and a susceptible strain (or species) influence the two-lineage community response to antibiotic exposure. Our model predicts that while increasing competition and antibiotics leads to increasing competitive release of the antibiotic-resistant strain, hitting a mutualistic community of cross-feeding species with antibiotics leads to a mutualistic suppression effect where both susceptible and resistant species are harmed. We next show that the impact of antibiotics is further governed by emergent spatial feedbacks within communities. Mutualistic cross-feeding communities can rescue susceptible members by subsidizing their growth inside the biofilm despite lack of access to the nutrient-rich and high-antibiotic growing front. Moreover, we show that antibiotic detoxification by resistant cells can protect nearby susceptible cells, but such cross-protection is more effective in mutualistic communities because mutualism drives mixing of resistant and susceptible cells. In contrast, competition leads to segregation, which ultimately prevents susceptible cells to profit from detoxification. Understanding how the interplay between microbial metabolic interactions and community spatial structuring shapes the outcome of antibiotic treatment can be key to effectively leverage the power of antibiotics and promote microbiome health.

## Author summary

Pathogens -microorganisms that make us sick- often live within dynamic and complex multispecies communities, where they may not only compete for limiting resources but also exchange beneficial resources or services with other resident species. While antibiotics are commonly used to get rid of such harmful microbes, the community-wide effects of antibiotic treatment and its consequences for antibiotic resistance are still not well understood. How do competitive or mutually beneficial interactions between antibiotic resistant and susceptible species influence community resistance to antibiotics? Here we investigate this question using a computational model. We find that antibiotic exposure favours the resistant lineage when resistant and susceptible strains are competitors but harms both types when they are mutualists. With antibiotic-detoxifying resistant cells, cross-protection of susceptible cells is more effective in mutualistic communities because mutualism drives mixing of susceptible and resistant cells. In contrast, competition leads to their segregation, precluding susceptible cells to profit from their competitor’s local detoxification. Our findings highlight that knowing not only what species are present but also how they interact with each other and arrange themselves in space is central to understanding antibiotic resistance and to informing the development of strategies that promote microbiome health.

## Introduction

The human body is home to extraordinarily diverse microbial communities, or microbiomes [1]. Metabolic interactions among microbial members are now known to play a critical role in host health, including beneficial effects such as protection against pathogens [2], but also detrimental effects such as obesity, diabetes, and enhanced virulence in polymicrobial infection sites [3–10]. When a pathogen arises within such diverse and dynamic ecosystems, recent evidence suggests that the efficacy of drug treatment not only depends on the target species and the drug treatment regimen used, but also on the other species present and on the nature of their interaction [11–16]. Key to controlling antibiotic resistance and managing microbiome health is therefore to understand which treatment strategy is most effective and under what conditions [17–19].

A major concern when using antibiotics is the potential emergence of *de novo* antibiotic resistant mutants and/or the competitive release of new or existing antibiotic resistant strains [19]. Competitive release–when one species or strain increases in density due to the decline in density of its competitors–is for instance one of the main causes of *C*. *difficile* infection, especially following treatment with broad-spectrum antibiotics. Such antibiotic therapy disrupts the normal gut microbiome composition, killing protective resident species, thus leading to the overgrowth of *C*. *difficile* [2]. Competitive release after drug treatment has also been demonstrated in rodent mixed strain malaria infections consisting of genetically distinct drug-resistant and drug-sensitive *Plasmodium chabaudi* clones [20–22], with the resistant strain rising in frequency due to the inhibition of its drug-sensitive competitor [20]. A key factor mediating the strength of competition between susceptible and resistant *P*. *chabaudi* strains is resource availability [23,24]. For instance, recent work has shown that resource abundance can lead to the competitive release of the resistant strain and increased virulence [23].

While there has been a strong focus on competition as a driver of antibiotic resistance and virulence, mutualistic and exploitative interactions among co-infecting bacteria have also been associated with enhanced virulence [4,25,26] and, in some cases, antibiotic resistance [11–14,27]. For example, Vega et al. (2013) showed that the pathogenic *S*. *typhimurium* was able to enhance its antibiotic tolerance by sensing indole produced by the commensal *E*. *coli* [12]. Another example of cross-species protection against antibiotics involves the beta-lactam susceptible *S*. *aureus*, which was protected from beta-lactam antibiotics when enclosed within a layer of resistant beta-lactamase producing *P*. *aeruginosa* [13]. In addition to cross-protection that arises when detoxifying enzymes are released into their local extracellular environment [13,28–30], recent studies have shown that cross-protection can also occur via intracellular detoxification [14,31,32]. For instance, Sorg et al. (2017) recently showed that chloramphenicol-resistant *S*. *pneumoniae* can protect chloramphenicol- susceptible *S*. *pneumoniae* by degrading chloramphenicol intracellularly, which then lowers the extracellular concentration of antibiotic in their neighbouring environment. Together, these examples highlight the importance of the interplay between species and strain metabolic interactions, their spatial arrangement, and the mode of resistance in shaping the outcome of antibiotic resistance. Although we use the terms strain and species interchangeably, we anticipate that competitive interactions will dominate among strains of the same species, whereas more diverse ecological interactions will be more common among species with more distinct metabolic profiles.

Top-down sequencing approaches have revealed important correlations between microbiome composition and host health (e.g., [1]), yet bottom-up approaches are indispensable for identifying the causal mechanisms underlying microbiome-mediated effects on their host. A major challenge of using a bottom-up approach, however, is that microbiomes are highly diverse resulting in large networks of microbial interactions that become substantially more complex as diversity increases. In order to make sense of such complexity, many studies—as the ones described above- have focused on more tractable, well-defined microbiomes with a reduced diversity and therefore smaller interaction networks. These studies have provided valuable insights into the causal links between microbiome structure and function and host health. For instance, previous work using two-species co-infection models have revealed the role of co-infection for increased virulence [4,9,33] and antibiotic tolerance [8,12,34]. Here we use a similar qualitative, two species model approach to develop an understanding of the basic principles of antibiotic perturbations on population structure.

Since microbes typically grow in multispecies, surface attached micro-colonies and biofilms [35,36], it is therefore key to understand the impact of species interactions and spatial arrangement on the dynamics of resistance [37,38]. Here we examine this idea by extending an established individual-based computer simulation model of bacterial biofilm growth on an inert surface [39]. Specifically, we investigate how the nature of the ecological relationship between antibiotic-resistant and sensitive strains or species across the conflict-mutualism continuum affects the community response to antibiotic treatment, and what is the role of spatial structure for such outcome.

## Results

### Community response to antibiotics is dependent on ecological interactions

**Fig 1:**
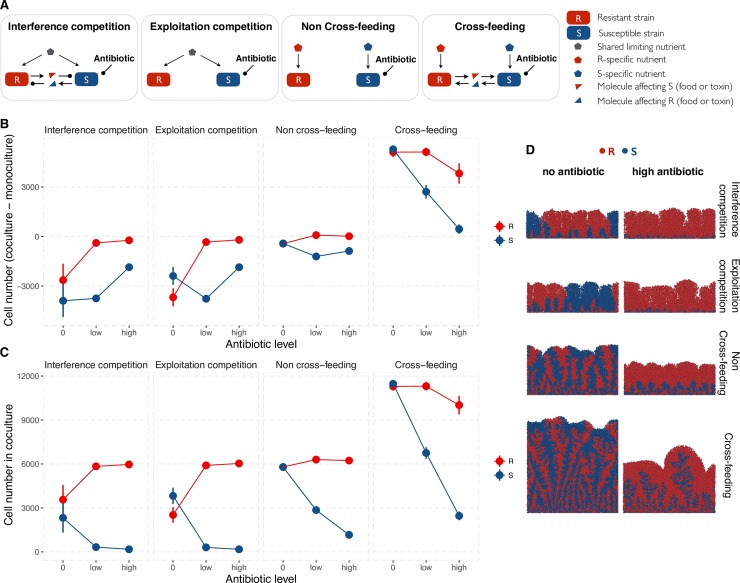
Antibiotic assault leads to the competitive release of the resistant lineage when susceptible and resistant cells are competitors but to a mutualistic suppression when they are mutualists. **A.** We consider four types of metabolic interactions between the resistant (R) and susceptible (S) strains. R and S compete for shared limiting nutrients and release a toxin that harms the other type (interference competition). R and S compete for shared limiting nutrients (exploitation competition). R and S may compete for space as they grow and divide but no direct competition for shared nutrients (non cross-feeding). R and S feed on each other metabolic by-products (cross-feeding). Open arrows represent a positive effect whereas oval arrows represent a negative effect upon the species they are pointing toward. **B.** For each scenario above, shown is the outcome of ecological interaction after 36h of growth either without (baseline) or with antibiotics. For this, we plot the number of R cells in coculture minus R cells in monoculture (R_co_ -R_mono_) and the number of S cells in coculture minus S cells in monoculture (S_co_—S_mono_). When above (below) the 0 dashed line, a strain does better (worse) in coculture than in monoculture. When R and S grow better together than alone, they are mutualists. When they grow worse together than alone, they are competitors. When one type grows better but the other grows worse, the former exploits the latter. **C.** Shown is the number of R and S cells after 36h of coculture growth. Competitive release of R occurs when *R*_co_ [antibiotic>0]—*R*_co_ [antibiotic = 0] > 0. Mutualistic suppression occurs when *R*_co_ [antibiotic>0]—*R*_co_ [antibiotic = 0] <0. Cross-species phenotypic resistance is defined as *S*_co_ [antibiotic>0]—*S*_mono_ [antibiotic>0] >0 (see S1 Fig for time series). **D.** The images show examples of simulations at t = 24h. Resistance is cost-free (assumption relaxed later). R and S are randomly seeded at 1:1.

To investigate the mechanistic and demographic underpinnings of community-mediated resistance in a spatial context, we implemented a mechanistically-explicit individual-based model consisting of an antibiotic-resistant (R) and a susceptible (S) species (or strain). Our model extends an established individual-based framework that simulates the growth and division of cells on an inert surface with explicit diffusion of nutrients and metabolites ([40], see Methods for further details on the model). Specifically, here we devise four distinct, metabolically-explicit, media that correspond to four distinct ecological relationships along the conflict-mutualism continuum, namely: “interference competition” where strains or species compete for shared limiting nutrients and release toxins that inhibit the growth of other species (e.g., bacteriocin production by, and toxic to, *E*. *coli* strains [41]); “exploitation competition” where there is competition for shared nutrients but no release of toxins; “non cross-feeding” where strains or species do not compete for shared nutrients and also do not release by-products; and “cross-feeding” where species release metabolic by-products that can be used by other species for growth (see Fig 1A for a schematic). As a baseline, we assume that all interactions are symmetric, that is, the nutrients consumed by resistant and by susceptible cells have the same nutritional value, and the toxins have the same inhibitory effect.

We first confirm that our metabolic interactions defined in Fig 1A lead to the expected ecological relationships, by contrasting the growth of each lineage in co-culture with growth in monoculture under equivalent drug and interaction environment (Figs 1B and S1). As expected, the interference and exploitation competition media generate strong competition (more growth in mono-culture than in co-culture). In the non-cross feeding medium, both species are weakly harmed by coculture growth despite the lack of competition for nutrients or the production of antimicrobials that harm the other partner, suggesting competition for space (Fig 1B). Such space competition arises from competition to gain access to the growing front of the biofilm where nutrient concentrations are highest. When the density of cells is low, competition for space is non-existent or very weak but it intensifies as the density of cells increases and the nutrient-rich front becomes more crowded. In contrast, cross-feeding leads to mutualism as seen by an increase in population densities when grown in coculture compared to when grown alone (Fig 1B).

We next ask: how does the type of ecological interaction between resistant and susceptible strains influence the community response to antibiotic perturbation? Generally, we find that the density of susceptibles decreases with increasing levels of antibiotics, regardless of their interaction with the resistant strain (Fig 1C). Whether the density of the resistant strain increases or decreases following antibiotic treatment, however, depends on whether susceptibles harm or help the resistant type. Specifically, we find that antibiotic perturbation leads to an increase in resistant cell densities when resistant and susceptible species are competitors (competitive release) but to a reduction in resistant density when they are mutualists (mutualist suppression, Fig 1B and 1C). Moreover, mutualistic cross-feeding weakens the negative impact of antibiotic exposure on the susceptible species—that is, susceptibles grow better in the presence of the resistant species than when alone, but this cross-species protection decreases with increasing antibiotic level (Figs 1B and 1C and S1). Note that cross-protection is purely measured by growth rates, and is therefore agnostic to the mechanism. As such, it does not imply a reduction of antibiotic inhibition, and can be due to an increase in intrinsic growth rate, due for instance to the supply of food by cross-feeding. In the non cross-feeding media, we see a weak competitive release of the resistant type, with the susceptible species doing worse when co-cultured with the resistant species than when alone, again due to competition for space (Figs 1B, 1C and S1). This negative effect of coculture growth on susceptibles is likely explained by the fact that resistant cells are able to invade the growing (nutrient-rich and high antibiotic) front first due to their growth advantage over susceptible cells whose growth is inhibited by antibiotics (Fig 1C and 1D). And as a result, the distance between the nutrient-rich front and susceptible cells gradually increases, ultimately leading susceptible cells to starvation.

In sum, our results suggest that antibiotic perturbation leads to the competitive release of the resistant species when the susceptible and resistant species are competitors and to the suppression of the resistant species when the two species are mutualists.

### Competitive release and mutualistic suppression are generally robust to costly resistance and to initial conditions

**Fig 2:**
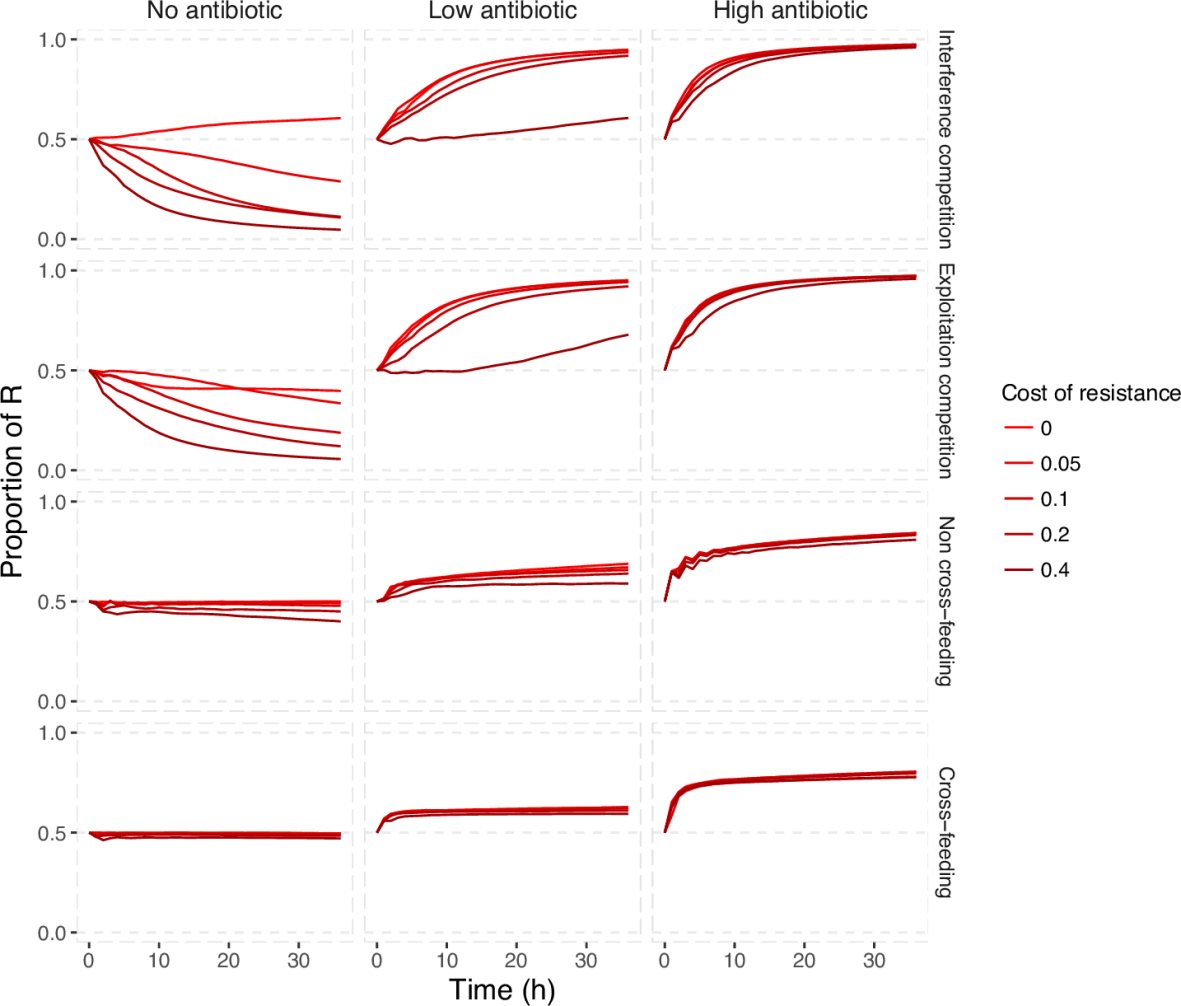
Costly resistance has minimal impact on competitive release and mutualistic suppression effects when antibiotics are present. The darker the red, the greater the cost of resistance. Here we assume that resistance costs are expressed as a reduction in maximum intrinsic growth rate (e.g., 0.1 indicates a 10% cost) (see Methods). See S2 Fig for plots showing coculture densities.

In the model presented above, we assume that there are no fitness costs of resistance. The no-cost scenario reflects cases where the resistant strain has either acquired compensatory mutations that alleviate the cost of resistance [42] or when resistance is intrinsic, in which case resistant and susceptible strains likely belong to different species [43]. But in many cases, resistance comes at a fitness cost, for instance, due to the acquisition of resistance via a plasmid or due to chromosomal resistance mutations with epistatic effects [44,45]. Adding cost of resistance to our model, we find that in the absence of antibiotics, the greater the cost of resistance and the strength of competition, the more quickly the susceptible strain outcompetes the resistant strain, as expected (Figs 2 and S2). In contrast, we find that the cost of resistance has little impact on resistant:susceptible ratios under strong antibiotic selection, and/or when the two types are mutualists (Figs 2 and S2).

The results in Fig 2 suggest that competitive release and mutualistic suppression of antibiotics are generally robust to costs of resistance (a result also robust to changes in inoculum size, S3 Fig). Next, we examine if they are also robust to variation in the initial configuration of the two species community. In an antibiotic-free environment, competitive communities are known to be more sensitive to initial conditions, while mutualistic communities are more robust [39]. Specifically, in a community with two equal competitors the most common species is usually favoured ([39]; see also S4A Fig). The reason for this positive frequency-dependent invasion effect is intuitive: all else equal, the species that starts at a higher frequency is more likely to dominate the nutrient-rich front, and cut off its competitor access to nutrients. In contrast, mutualistic community composition is robust to initial conditions, as partner co-dependency generates negative frequency dependence [39].

How does applying antibiotics influence competition-mediated sensitivity and mutualism-mediated robustness to initial conditions? Unlike in our simple equal competitor scenario without antibiotics, applying antibiotics to the competitive community switches the system to a state where the resistant species is generally favoured independently of initial proportions and spatial distribution (S4A Fig). This result suggests that the competitive release of a resistant species following an antibiotic perturbation is stable to varying initial demographic conditions (S4 Fig). Note that this effect occurs because the resistant and sensitive strains have equal competitive abilities (resistance is cost-free). In the more biologically expected scenario of resistance being costly, the sensitive strain would be favoured at no or low antibiotics. But as the antibiotic concentration increases and passes some threshold, the benefits of being resistant outweigh its costs, and as such, the resistant strain would now be favoured over the sensitive strain. Cross-feeding (and mutualistic) communities, however, continue to show a robust signature of negative frequency dependence (i.e. the rare species is favoured), reaching a stable equilibrium proportion of susceptibles that is independent of initial proportions and intermixing but depends on the antibiotic concentration (lower proportion of sensitives for higher antibiotic concentrations) (S4A Fig). Moreover, despite a surface blanket of resistant cells (Fig 1D), susceptible cells remain generally intermixed with resistant cells (S4B Fig) and their densities remain positively correlated irrespective of initial proportions and intermixing, thereby suggesting that the suppression of mutualists is also robust to varying initial demographic conditions.

### Spatial mixing of mutualists enhances cross-protective detoxification

So far we have assumed that antibiotic resistance in a focal cell has no impact on the abundance of antibiotic encountered by other cells. However, resistance often occurs through antibiotic degradation, leading to a reduction in the levels of antibiotics present in the local environment of a focal resistant cell (i.e. detoxification) [14,31,32,46]. In this context, the presence of antibiotics can change the nature of an ecological relationship between species, for example turning a resistant lineage from competitor to protector [31,38,47]. Next, we test the impact of protective detoxification by extending our model to allow resistant cells to remove the antibiotic through a process that mimics intracellular enzymatic degradation (consistent with typical periplasmic beta-lactamase activity or with cytoplasmic antibiotic-modifying or -degrading enzymes, there is no release of antibiotic degrading enzyme; see Methods) [14,32]. Such process leads to the detoxification of the environment surrounding the antibiotic detoxifying resistant cells, thereby benefiting any nearby susceptible cells.

**Fig 3:**
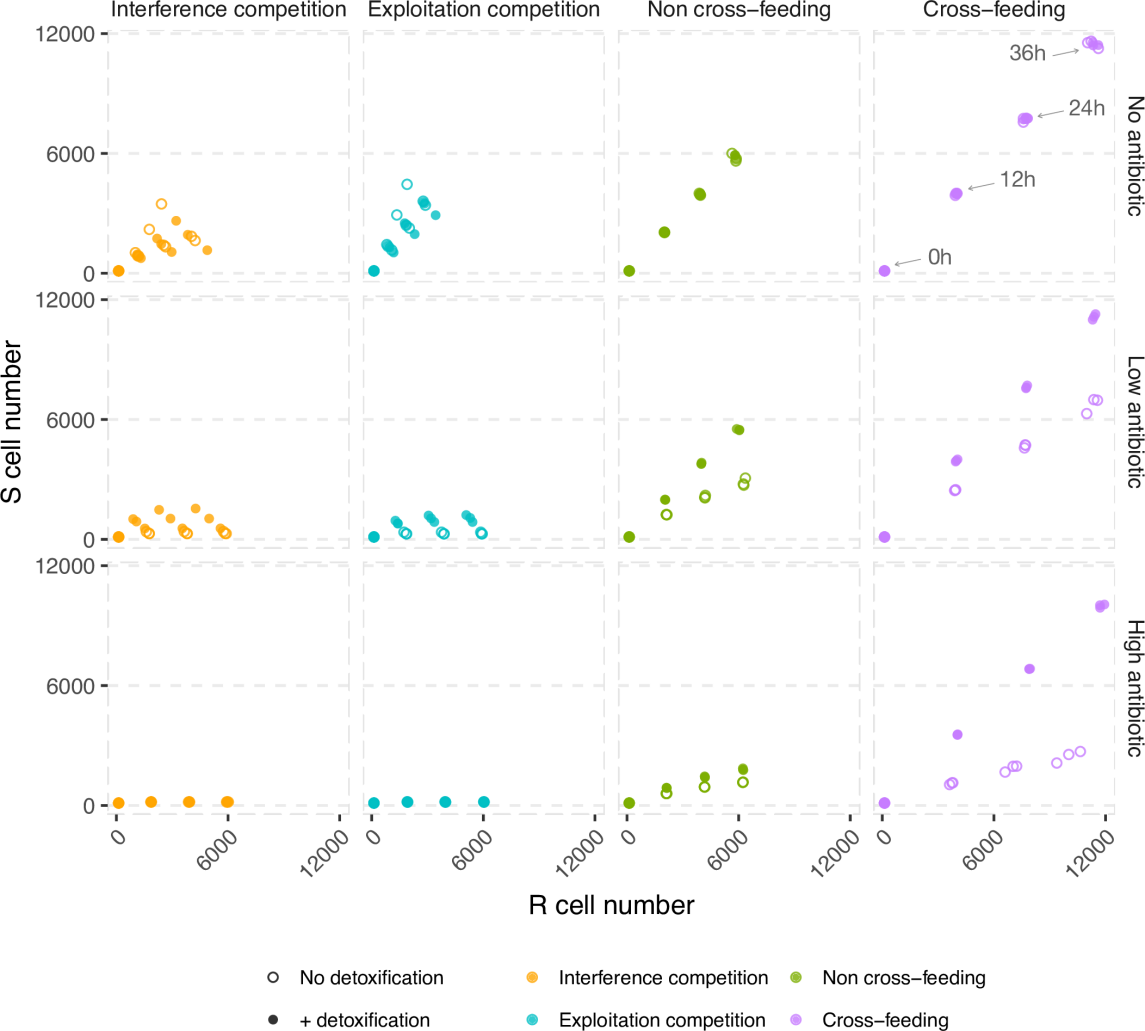
A synergistic interaction between cross-feeding and antibiotic detoxification further enhances community resistance to antibiotics. Plotted are the coculture densities of susceptible and non-detoxifying resistant cells (no detoxification), or of susceptible and detoxifying resistant cells (with detoxification) at different times of colony growth (t = 0h, t = 12h, t = 24h, and t = 36h). As an example, the time points are labeled for the cross-feeding and no antibiotic case (top right panel). Note that although irrelevant for the ‘+ detoxification’ case, the top row is included in the figure to show the ‘no antibiotic’ baseline. The two species are seeded randomly and at 1:1. These results are robust to costs of resistance (S6 and S7 Figs).

**Fig 4:**
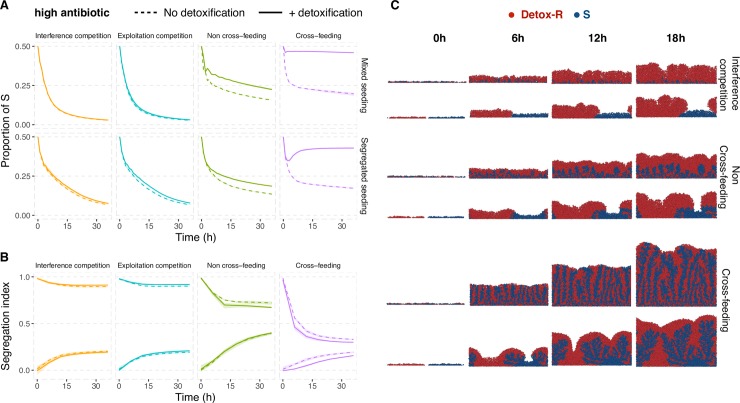
Cross-feeding drives mixing, allowing susceptible cells to benefit from the antibiotic detoxification by neighbouring resistant cells. **A.** S cells grow in coculture with non-detoxifying R cells (dashed line) or with antibiotic-detoxifying R cells (filled line) under high antibiotic conditions. The two types are seeded either in a randomly mixed or segregated manner (see **C** for images of seeding at t = 0h). **B.** Segregation index of communities shown in **A** when seeded in a randomly mixed (bottom line, with segregation index ~0 at t = 0) or segregated (top line, with segregation index ~1 at t = 0) manner (for segregation index description, see Methods). Mutualistic communities generally tend to mix while competitive communities tend to segregate. The two ‘alternative’ outcomes of segregation pattern observed for the competition and non cross-feeding cases occur because the potential for species mixing or segregation depends on a lineage ability to grow. This means that, if the susceptible cells start mixed and are unable to grow- such as with high antibiotics, they will inevitably remain mixed as they cannot grow into segregated clusters of susceptible cells. **C.** Images show representative examples of simulations from one of the scenarios represented in **A** at t = 0h (seeding) and after 6h, 8h, 12h, and 18h growth for the case when the resistant strain is detoxifying (see also S1–S4 Movies).

Previous work has shown that competitors generally tend to segregate as they grow and divide while mutualists tend to mix ([39,48,49]; and also S4B Fig). Therefore, we predict that cross-protection will be more effective in mutualistic communities because mutualism drives mixing of susceptible and resistant cells, allowing the susceptible cells to benefit from their partner’s local detoxification. In contrast, because competition leads to species segregation, such spatial separation will limit the ability of susceptible cells to profit from their competitor’s local detoxification. Consistent with our prediction, we find that detoxification by the resistant lineage provides the greatest rescue effect for susceptible cells when the lineages are engaged in mutualistic exchange (Fig 3), leading to greater intermixing (Fig 4). Detoxification coupled with cross-feeding leads to a reduction in the concentration of antibiotics within the biofilm to levels much lower than those reached in other media (S5 Fig). This occurs because antibiotic degradation is growth-dependent, and as such the growth-promoting effect of cross-feeding leads to further antibiotic detoxification. Put another way, susceptibles feed their detoxifier resistant partner, and in turn, benefit from not only increased food provision but also increased detoxification. When resistant and susceptible species are competitors, however, antibiotic detoxification is not sufficient for susceptibles to persist and they are quickly outcompeted (Fig 3). Even in the non cross-feeding medium, where susceptibles are able to persist for longer, their proportion decreases slowly with time (Fig 3). And because of their unavoidable growth disadvantage compared to resistant cells, we expect that without a mechanism to drive species mixing, susceptible cells will quickly be buried and starved at the bottom of the biofilm.

To further test the impact of spatial patterning, we ran a new set of simulations where the resistant and susceptible types are now initially segregated. We predict that spatial segregation should accelerate the decline of non cross-feeding susceptibles because there are no mutual benefits driving the mixing of susceptible cells with detoxifying resistant cells, but only the exploitation of detoxification by susceptibles. Consistent with this prediction, we find that in the non cross-feeding media, the proportion of susceptibles initially decreases more quickly when resistant and susceptible cells start segregated (Fig 4A). But in the cross-feeding media, despite a decrease in the proportion of susceptibles at the start of the experiment, susceptible cells mix with their detoxifying partner and reach a stable equilibrium proportion (Fig 4A–4C). This supports the idea that, in structured communities, the mutual benefits from cross-feeding are crucial to drive species mixing and allow susceptible cells to exploit the antibiotic detoxification of their neighbouring resistant cells. Furthermore, we can see that when susceptible and resistant species are competitors, the proportion of susceptible cells declines more slowly when they are initially segregated from resistant cells than when mixed, and likely occurs because spatial segregation weakens competition (Fig 4A).

## Discussion

Microbes live in metabolically-connected and spatially extended multispecies communities [35]. Understanding how microbial communities respond to antibiotic assault is therefore central to improving human health. Our model suggests that antibiotic perturbation can lead to the competitive release of resistant competitors, or to the mutualistic suppression of resistant partner species–with the outcome tuned by costs of resistance, spatial patterning, and potential for detoxification. How can these findings inform the development of strategies that aim at promoting microbiome health? Crucially, whether one wants to enhance or attenuate competitive release and/or mutualistic suppression depends on the species that are present and the impact they have on their host, that is, whether they help or harm their host [37]. Target infections are commonly polymicrobial [8,33,50], particularly for chronic infections such as in cystic fibrosis lung infections [51] or chronic wounds [52]. Even for acute and clonal infections, the antibiotic administration still has a strong community context due to impacts on commensal microbiomes, often accompanied by unintentional collateral harm to the host [53,54].

Competitive release occurs when two species compete for shared limiting resources and the removal of one species liberates resources that can be used by the other species, which then increases in density. Because of higher density, the probability of getting mutations that further improve the fitness of the resistant strain increases, potentially leading to higher rates of resistance [17]. The role of spatial competition for competitive release and the spread of drug resistance has recently been studied experimentally in microbial colonies [55]. In the absence of antibiotics, *de novo* resistant clones may remain trapped and starved within the inner region of the colony, layered with growing sensitive cells. When high antibiotic concentrations are applied, however, the sensitive cells are killed and eventually removed from the growing front, thus freeing space and resources that can then be taken up by the resistant cells. A similar finding has been observed in the context of chemotherapy using a computer simulation of tumor growth [56]. We can therefore see how competitive release is a major concern for health if the resistant strain is a pathogen, as such release may facilitate the rise of antimicrobial-resistant parasites and virulence [17,20,57]. Under such conditions, the medical priority is therefore to reduce the rise of resistant strains via competitive release. Two candidate mechanisms for preventing the emergence of antibiotic resistance under such competitive scenario include maintaining competitive suppression, that is ensuring that cells competing with resistant cells are not inhibited or killed by the antibiotic, and/or targeting resistant cells only, for instance by combining antibiotics with phage therapy [58,59].

Although ubiquitous, microbial competition is not universal [36]. The oral and gut microbiome, for instance, are replete with species that benefit from the presence of other microbial species [3,4,60–63]. Within a polymicrobial infection context, examples are growing where co-infecting bacteria enhance each others’ growth [8,26,33]. Such dense, mutualistic communities are of particular concern for controlling infections as their higher densities may hinder clearance of targeted infections, and also, increase the likelihood of emergence of antibiotic-resistant mutants. So what are the consequences of disrupting communities of antibiotic-resistant and susceptible mutualists? As antibiotic concentration increases, we find that the density of the susceptible species decreases, causing the decline of the resistant species (mutualist suppression), but despite such decline in density, the susceptible species grows better in coculture than in monoculture, illustrating a continued impact of the mutualistic exchange (cross-species phenotypic resistance). From a medical intervention perspective, this means that one can knock down resistant bacteria by hitting mutualistic susceptible species, but also, that one can protect susceptible bacteria by not hitting resistant species that support their growth. If the susceptible species is a pathogen, such cross-species protection is therefore likely to reduce the efficacy of the antibiotic treatment.

The potential for susceptible cells to be protected against antibiotics is in principle enhanced when resistant bacteria can detoxify their environment. Empirical evidence of cross-protection of susceptible cells by antibiotic-resistant detoxifying cells is accumulating in the literature, and has been documented both within the same species [14,28,64–67] and between different species [30,68], and also in different contexts, including cases in which the antibiotics were produced by other community members [69] or exogenously added to the growth medium [14,28,31,64–66,68]. Our work shows that, in spatially extended environments, the emergent spatial arrangement of resistant and susceptible cells influences greatly whether susceptible cells can benefit from detoxification. Importantly, our results suggest that this protective effect is much more effective in mutualistic communities. The reason for this effect is that mutualistic partners tend to spatially mix, thus allowing susceptible cells to fully benefit from the detoxification by their spatially proximate mutualistic partner.

In contrast, competition leads to segregation, which ultimately prevents susceptible cells to profit from detoxification. This finding is in line with previous work on the evolution of cooperation in microbial biofilms showing that competition leads to the formation of clonal groups (high segregation) that insulates enzyme-secreting strains from non-secreting strains, thus precluding non-secretors from receiving the benefits of the secreted products [70,71]. Our model assumes that the antibiotic slows down the growth of susceptible cells (bacteriostatic). Generally, we expect the effect of protective detoxification to be stronger in the presence of bacteriostatic than bactericidal (killing) antibiotics. This is because, with bacteriostatic antibiotics, most cells will eventually be able to resume growth once the concentration of antibiotic drops to levels low enough to permit growth. In contrast, with bactericidal antibiotics, only the cells that survive the antibiotic assault will be able to grow. But this killing effect will be reduced in communities where susceptible and resistant lineages intermix, and so less relevant in mutualistic communities. Although our simulations do not look at the effect of bacteriocidal antibiotics, it would be interesting to test these ideas both theoretically and experimentally.

Our results are based on the assumption that the antibiotic is applied at time 0, thus before any interaction between resistant and susceptible cells has taken place. In a clinical context, however, antibiotics will likely be applied to an already established microbial community with a given ecological and spatial structure that reflects the no antibiotic case. How does the timing of antibiotic administration impact our results? We ran a new set of simulations where the antibiotic is now added after 4h, 12h, or 24h of biofilm growth, and found that adding antibiotics at later stages of biofilm development generally favours the susceptible strain, and can even prevent the competitive release of the resistant strain (S8 Fig). This positive effect of delayed antibiotic administration on the susceptible lineage was stronger at low levels of antibiotics and with detoxification by resistant cells, as expected.

Our model assumes that all the interactions are symmetric. Any deviations from the baseline dynamics are therefore due to the effect of the antibiotic and/or the cost or resistance. Interactions between resistant and susceptible strains may, however, be asymmetric, potentially changing the benefits and costs of interactions. How can asymmetries impact the outcome of antibiotic treatment? When resistant and susceptible species are mutualists, their interests are largely aligned. As such, the cross-feeding and cross-protection benefits received by susceptible cells depend on the growth of its mutualist resistant partner. If susceptibles start outgrowing the resistant type, the reciprocal benefits of mutualism are diminished, and this will ultimately harm susceptibles due to the lower provision of food and lower detoxification by resistant cells. This negative feedback can help stabilize the mutualistic interaction, and consequently, the response to antibiotic treatment.

Clearly, hosts play a crucial role in shaping the composition and structure of their microbiomes, not only by providing shelter and food to their resident microbes, but also by producing antimicrobial cells and molecules that inhibit or kill potential enemies [72]. In turn, microbes affect their host’s fitness and behaviour in various ways, including aid with digestion and supplementation of essential nutrients [73], as well as protection from pathogens, either directly through competition, or indirectly by eliciting the host’s immune response [74,75]. While a considerable amount of work has been done to understand how within-host community dynamics shape host health, including virulence evolution and drug resistance, the majority of these studies have focused on interactions between parasites and in competition. Our work suggests that broadening our view of microbe-microbe and host-microbe interactions to include the full conflict-mutualism spectrum is important to elucidate the causes and consequences of intra- and interspecific interactions in host health.

Our work focuses on a two-strain or two-species microbial community living in a simple environment (one/two resources and a single antibiotic), which is undeniably an oversimplified view of natural microbial ecosystems. Although our results are likely not generalizable to the large diversity of microbiomes, such minimal microbiome approach allows us to identify testable principles of community-mediated antibiotic resistance which can lay the foundations for further research on more complex communities. For instance, it would be interesting to extend our model to investigate the outcome of antibiotic treatment in a community consisting of isogenic resistant and susceptible strains plus a third resistant or susceptible strain that acts as a mutualist or competitor. Testing these ideas in more diverse communities and complex environments will help elucidate both general and system-specific principles that determine the outcome of antibiotic therapy.

In sum, our results suggest that the interplay between the metabolic and spatial relationships of resistant and susceptible strains within a community plays an important role in shaping the outcome of antibiotic treatment. Understanding this relationship can therefore be key to develop effective control strategies. We expect that the spatial segregation and lower density of competitive communities should facilitate the clearing of an infection because the target sensitive species is isolated from the resistant species, and as such, more vulnerable to antibiotic clearance. In such competitive scenario, a priority is to maintain competitive suppression, and therefore using narrow-spectrum antibiotics may be more effective than broad-spectrum antibiotics. In contrast, the spatial mixing and higher densities of mutualistic communities will make it harder to clear the target species. Under such mutualistic conditions, narrow-spectrum and bacteriostatic antibiotics may therefore be less effective as cross-protection and cross-feeding increase the likelihood that sensitive cells will be able to resume growth once the concentration of the bacteriostatic antibiotic falls below levels permissive for growth. One potential treatment strategy for the control of mutualistic communities would be to first disrupt the mutualism, e.g. through a diet change that induces a shift in metabolic interaction [76], to lower mixing of resistant and susceptible cells, thus facilitating clearance. Testing these ideas experimentally would be an important step towards effectively leveraging the power of antibiotics to promote microbiome health.

## Methods

### Individual based model description and assumptions

Our model is an extension of an established, empirically tested individual-based framework that simulates the growth and division of cells on an inert surface. This modelling framework has been used extensively over the past decade to understand the behaviour, ecology, and evolution of microbial communities, including studies looking at the drivers of genotypic spatial segregation in biofilms and its consequences for within-species [70,77] and between-species [71] cooperation, the evolution of quorum sensing in bacterial biofilms [78], the interplay between food for detoxification mutualisms and species mixing [39], the costs and benefits of microbial adhesion within communities [79], and mechanisms of host control of the microbiome [80,81]. For a detailed description and justification of the assumptions of this modelling framework, see [40,82,83]. Briefly, the model assumes that cells are spheres that sense and are affected by the local concentration of solutes. The solute concentrations vary through space due to Fickian diffusion and to the reaction dynamics mediated by each cell. The solute concentrations are assumed to be in pseudo steady-state relative to biomass growth (see [84] for the timescale discussion). More specifically, cells grow by consuming nutrients that diffuse from a well-mixed bulk liquid compartment located above the biofilm. Within that compartment, nutrients are available at a fixed concentration, and diffuse at a fixed rate through the biofilm (Table 1). Nutrient gradients are created as a result of nutrient consumption. Cell growth rate depends on local nutrient concentrations and follows Monod kinetics (see Tables 1 and 2). Cells grow and then divide, giving rise to two daughter cells that push apart neighbouring cells. Within the biofilm, cells interact directly by physically shoving for space during growth [40] and indirectly through chemical reactions, such as the consumption of nutrients and other metabolically-mediated interactions (see Table 2 for a description of the biochemical processes involved in our simulations for each of the media studied). In particular, our model assumes that cells can release antimicrobial toxins that inhibit the growth of other strains or species (interference competition media) or release growth-promoting metabolic by-products (cross-feeding media). In addition, our model focuses on a two-species (or two-strain) community consisting of an antibiotic-resistant (R) and a susceptible (S) species (strains). The growth of susceptible cells is inhibited by a narrow-spectrum antibiotic (bacteriostatic) following a simple inhibition but not the growth of resistant cells. As for the nutrients, the antibiotic is present at a fixed concentration within the bulk liquid compartment, and diffuses at a fixed rate into the biofilm (Table 1). We start by assuming that resistant cells do not inactivate the antibiotic (no detoxification), which can be seen as a type of intrinsic resistance where the strain lacks a target for that specific antibiotic [85] or a scenario where the enzyme remains locked into the periplasm [64]. We later relax this assumption by considering that resistant cells detoxify their local neighbourhood, and as a consequence, nearby susceptible cells benefit from a more detoxified environment. Antibiotic detoxification occurs simultaneously with nutrient consumption, and therefore without growth, there is no detoxification (Table 2). Also, there is no release of antibiotic-degrading enzyme into the external environment thus implicitly mimicking a scenario where the enzyme is located in the outer-membrane [64].

**Table 1:** Parameters used in the simulations.

Symbol	Description	Value	Unit
*μ*_*max*_	Maximum growth rate of strain R (S)	1*	h^-1^
*c*	Cost of antibiotic resistance	0 or as indicated	h^-1^
*K*_*N*_	Half-saturation constant for growth on substrate N	3.5 x 10^−5^*	g. L^-1^
*K*_*E*_	Half-saturation constant for growth on substrate E	3.5 x 10^−5^*	g. L^-1^
*Y*_*N*_	Biomass yield per N consumed	0.5*	g. g^-1^
*Y*_*E*_	Biomass yield per E consumed	0.5*	g. g^-1^
*Ki*_*T*_	Toxin inhibitory constant	0.1	g. L^-1^
*Ki*_*A*_	Antibiotic inhibitory constant	0.1	g. L^-1^
N	Concentration of substrate N in the bulk liquid environment (constant throughout simulations)	0.05	g. L^-1^
A	Concentration of antibiotic in the bulk liquid environment (constant throughout simulations)	0 or: 0.05 (low toxicity) 0.2 (high toxicity)	g. L^-1^
T_s_	Local extracellular concentration of toxin released by S and inhibiting R	NA	g. L-1
T_R_	Local extracellular concentration of toxin released by R and inhibiting S	NA	g. L-1
E_s_	Local extracellular concentration of by-product released by S and used by R for growth	NA	g. L-1
E_R_	Local extracellular concentration of by-product released by R and used by S for growth	NA	g. L-1
*α*	Antibiotic detoxification	0 or as indicated	g. g^-1^
*D*_*N*_	Diffusivity of nutrient	7.2 x 10^−6^	m^2^. day^-1^
*D*_*E*_	Diffusivity of by-product	7.2 x 10^−6^	m^2^. day^-1^
*D*_*A*_	Diffusivity of antibiotic	7.2 x 10^−6^	m^2^. day^-1^
*D*_*T*_	Diffusivity of toxin	7.2 x 10^−6^	m^2^. day^-1^

*Values from [71]

**Table 2:** Reactions and respective stoichiometry of biological processes used in the simulations.

Process	Solute								Biomass		Rate expression
	*N*	*N*_*R*_	*N*_*S*_	*E*_*R*_	*E*_*S*_	*T*_*R*_	*T*_*S*_	*A*	*X*_*R*_	*X*_*S*_	
**Exploitation competition media**											
R growth	-1/Y_N_							-α	1		$(\mu_{max}-c)\frac{N}{N+K_{N}}X_{R}$
S growth	-1/Y_N_									1	$\mu_{max}\frac{N}{N+K_{N}}\frac{{Ki}_{A}}{A+{Ki}_{A}}X_{S}$
**Interference competition media**											
R growth and toxin production	-1/Y_N_					1		-α	1		$(\mu_{max}-c)\frac{N}{N+K_{N}}\frac{{Ki}_{T}}{T_{S}+{Ki}_{T}}X_{R}$
S growth and toxin production	-1/Y_N_						1			1	$\mu_{max}\frac{N}{N+K_{N}}\frac{{Ki}_{A}}{A+{Ki}_{A}}\frac{{Ki}_{T}}{T_{R}+{Ki}_{T}}X_{S}$
**Non cross-feeding media**											
R growth		-1/Y_N_						-α	1		$(\mu_{max}-c)\frac{N_{R}}{N_{R}+K_{N}}X_{R}$
S growth			-1/Y_N_							1	$\mu_{max}\frac{N_{S}}{N_{S}+K_{N}}\frac{{Ki}_{A}}{A+{Ki}_{A}}X_{S}$
**Cross-feeding media**											
R growth from consuming N with by-product production		-1/Y_N_		1				-α	1		$(\mu_{max}-c)\frac{N_{R}}{N_{R}+K_{N}}X_{R}$
R growth from consuming S by-products with by-product production				1	-1/Y_E_			-α	1		$(\mu_{max}-c)\frac{E_{S}}{E_{S}+K_{E}}X_{R}$
S growth on N with by-product production			-1/Y_N_		1					1	$\mu_{max}\frac{N_{S}}{N_{S}+K_{N}}\frac{{Ki}_{A}}{A+{Ki}_{A}}X_{S}$
S growth on by-products of R with by-product production				-1/Y_E_	1					1	$\mu_{max}\frac{E_{R}}{E_{R}+K_{E}}\frac{{Ki}_{A}}{A+{Ki}_{A}}X_{S}$

**Note**: c = 0 when there is no cost of resistance and α = 0 when antibiotic detoxification is turned off.

For the full list of symbols used in the model and specific parameters used in the simulations, see Table 1. Other simulation parameters based on previous work are described in detail in [39]. Unless otherwise stated, all simulations are seeded at t = 0h with 120 cells of each species, thus giving a total of 120 cells in monoculture and 240 cells in coculture. The plots show the mean of 3 independent simulations.

### Segregation index

The segregation index calculation used here has been described in detail in [39] and is adapted from the methodology used in previous work to measure population segregation in biofilms [71]. Briefly, the segregation index of the resistant type (*s*_*R*_) in a community consisting of resistant and susceptible cells indicates the degree to which resistant cells are spatially segregated from susceptible cells, and is measured as

$$s_{R}=(p_{R\_local}-p_{R\_global})/(1-p_{R\_global})$$

where *p*_*R_local*_ is the proportion of resistant cells within their local neighbourhood and *p*_*R_global*_ is the proportion of resistant cells in the whole population. Thus, the closer *s* is to 0 the more mixed (randomly distributed) S and R cells are within the community, whereas the closer *s* is to 1 the more segregated (or assorted) they are. In addition, the proportion of R cells in their local neighbourhood is calculated as follows. For each individual cell (*c*_*i*_) of type R in a population of *N* = *N*_*R*_ + *N*_*S*_ cells, we identify all neighbour cells (*c*_*j*_) falling within a 10μm radius neighbourhood distance and define the proportion of cells identical to *c*_*i*_ in the neighbourhood of *c*_*i*_ as:

$$p_{local}\;(c_{i})=\frac{1}{N_{d}}\sum_{j=1}^{N_{d}}g(c_{i},c_{j})$$

where *N*_*d*_ is the number of cells falling within a distance of 10 μm, and where *g*(*c*_*i*_, *c*_*j*_) = 0 if *c*_*i*_ and *c*_*j*_ belong to different types or *g*(*c*_*i*_, *c*_*j*_) = 1 if *c*_*i*_ and *c*_*j*_ belong to the same type. The proportion of resistant cells in their local neighbourhood is then defined as:

$$p_{R\_local}=\frac{1}{N_{R}}\sum_{i=1}^{N_{R}}p_{local}(c_{i}).$$

## Supporting information

S1 Fig Timeseries of simulations shown in Fig 1. Antibiotic assault leads to strong competitive release of the antibiotic-resistant strain when the susceptible strain is a strong competitor (interference competition and exploitation competition media), to weak competitive release of the antibiotic-resistant strain when susceptibles are weak competitors (non cross-feeding media), but to a reduction in resistant density when they are mutualists (mutualist suppression) (cross-feeding media).
(PDF)

S2 Fig Impact of cost of resistance on the coculture densities of R and S. Shown are the densities of R (red) and S (blue) in coculture and the sum (grey). We can see that cost of resistance has a strong effect on coculture densities when R and S are competitors and antibiotics are absent, but little to no impact when antibiotics are present or when R and S are cross-feeding mutualists. Here we consider that costly resistance leads to a reduction in the maximum intrinsic growth rate (e.g. 0.1 indicates a 10% cost) (see Methods and Table 2).
(PDF)

S3 Fig How communities respond to antibiotics is robust to varying the inoculum size. Simulations are seeded 1:1 with 60, 120 (default), or 960 cells of each type. Biofilms are grown for 36h. Resistance is non-costly or costly (c = 0.2).
(PDF)

S4 Fig Competitive release and mutualistic suppression are robust to variations in initial proportion and initial spatial distribution. Here we vary both the initial proportion of S cells (5%, 50%, or 95%) and the degree of mixing between S and R cells (mixed, filled line; or segregated, dashed line) at inoculation. **A.** We can see that, in the interference competition medium, the most common type wins (positive frequency-dependent selection) when no antibiotics are present, but applying antibiotics to the medium shifts the balance towards resistant species being favoured irrespective of initial conditions. In the cross-feeding medium, however, susceptibles merge towards an equilibrium proportion that is independent of initial proportions but that decreases with increasing level of antibiotics. **B**. Segregation index of communities shown in S4A. See Methods for segregation index description.
(PDF)

S5 Fig Nutrient and metabolite concentrations within the biofilm. Shown are representative profiles of the average of solute concentrations (nutrients, by-products, toxins, and antibiotic) as a function of community height after 24 hours of growth. The gray horizontal dashed line shows the mean of the height of the biofilm. The two species are seeded randomly and at 1:1.
(PDF)

S6 Fig Coculture densities of R and S for varying cost of resistance without or with antibiotic detoxification. Plotted are the densities after 36h of coculture growth. At inoculation, the two types are seeded randomly and at 1:1.
(PDF)

S7 Fig Proportion of resistant cells for varying cost of resistance and detoxification level. The two types are seeded randomly and at 1:1.
(PDF)

S8 Fig Timing of antibiotic administration can impact outcome. Antibiotics are added at 0h (default), or after 4h, 12h, or 24h of biofilm growth. Shown are the coculture densities of R and S for every 4 hours from the time the antibiotic is added to 36h of biofilm growth in total (ie., growth without antibiotics plus growth with antibiotics). We can see that later antibiotic addition favours susceptibles, and can even prevent the competitive release of the resistant strain. This effect is stronger with detoxification and when antibiotic concentrations are low. In the simulations, resistance is costly (c = 0.2).
(PDF)

S1 Movie Non cross-feeding and no antibiotic detoxification conditions. Video showing that without cross-feeding, the resistant (red) and susceptible (blue) cells remain spatially segregated. Related to Fig 4.
(MOV)

S2 Movie Cross-feeding and no antibiotic detoxification conditions. Video showing cross-feeding between resistant and susceptible cells drives species mixing. Related to Fig 4.
(MOV)

S3 Movie Non cross-feeding and with antibiotic detoxification conditions. Video showing that without cross-feeding, there are no mutual benefits driving the mixing of susceptible cells with detoxifying resistant cells, therefore precluding susceptible cells to fully profit from antibiotic detoxification. Related to Fig 4.
(MOV)

S4 Movie Cross-feeding and with antibiotic detoxification conditions. Video showing that cross-feeding coupled with antibiotic detoxification provides the greatest rescue effect for susceptible cells. Related to Fig 4.
(MOV)
