# Supplementary figures and images for: Community interactions and spatial structure shape selection on antibiotic resistant lineages
Source: PLoS Comput Biol. 2018 Jun 21;14(6):e1006179. doi: 10.1371/journal.pcbi.1006179 (PMC6013025; doi:10.1371/journal.pcbi.1006179)

# S1 Figure

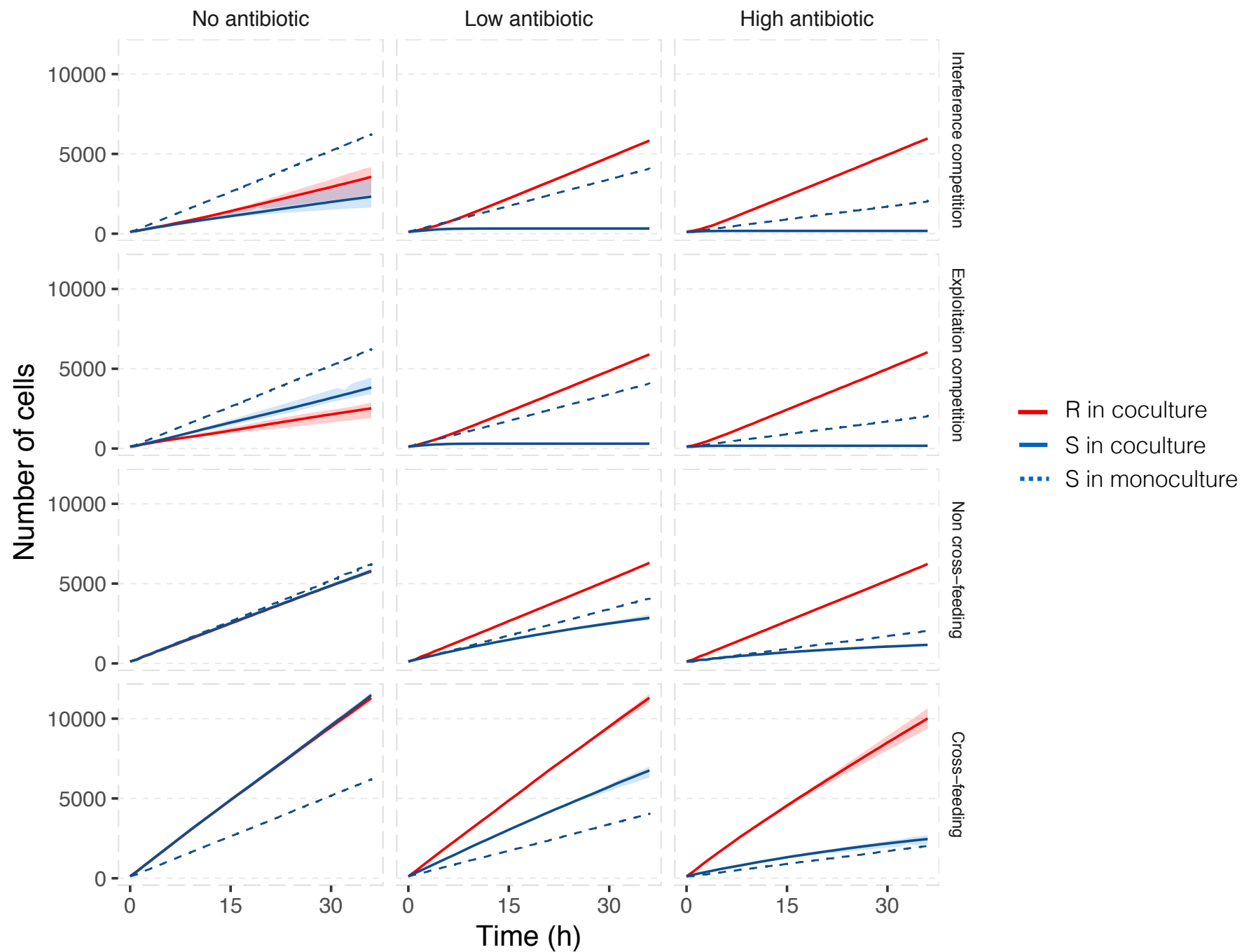

Supplement: S1 Fig — Antibiotic assault leads to strong competitive release of the antibiotic-resistant strain when the susceptible strain is a strong competitor (interference competition and exploitation competition media), to weak competitive release of the antibiotic-resistant strain when susceptibles are weak competitors (non cross-feeding media), but to a reduction in resistant density when they are mutualists (mutualist suppression) (cross-feeding media). (PDF) [file pcbi.1006179.s001.pdf]

# S2 Figure

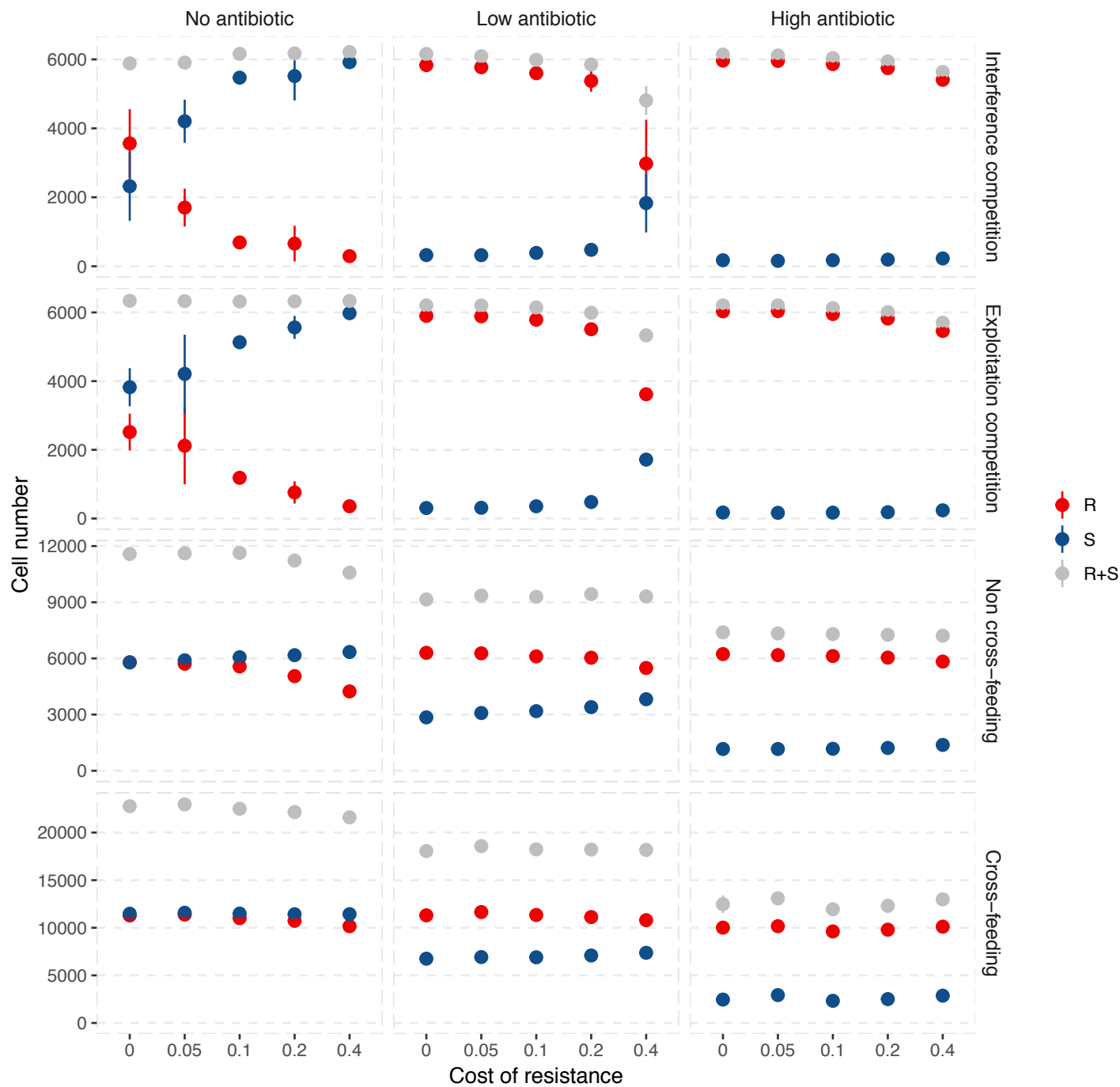

Supplement: S2 Fig — Shown are the densities of R (red) and S (blue) in coculture and the sum (grey). We can see that cost of resistance has a strong effect on coculture densities when R and S are competitors and antibiotics are absent, but little to no impact when antibiotics are present or when R and S are cross-feeding mutualists. Here we consider that costly resistance leads to a reduction in the maximum intrinsic growth rate (e.g. 0.1 indicates a 10% cost) (see Methods and Table 2). (PDF) [file pcbi.1006179.s002.pdf]

# S3 Figure

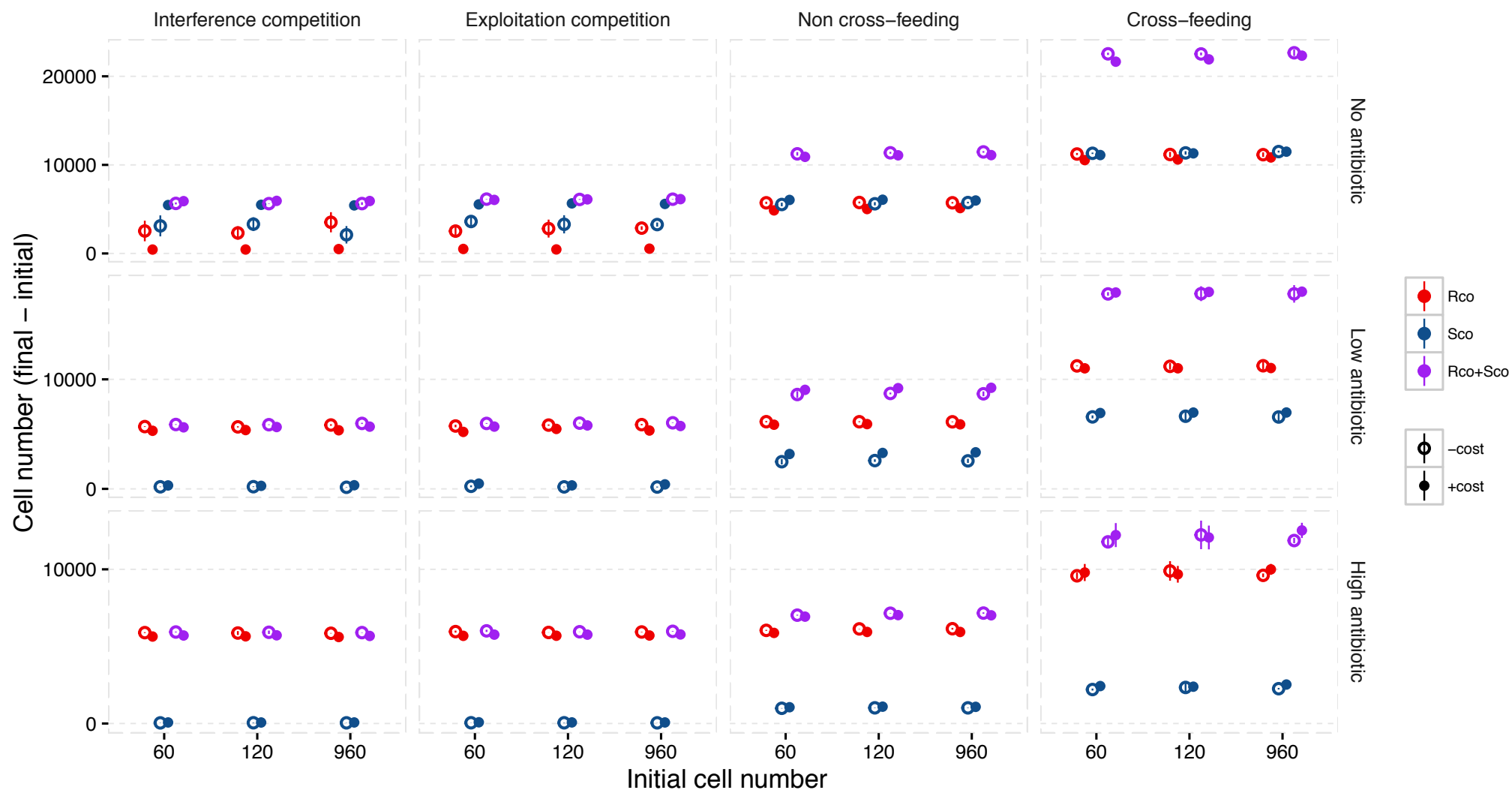

Supplement: S3 Fig — Simulations are seeded 1:1 with 60, 120 (default), or 960 cells of each type. Biofilms are grown for 36h. Resistance is non-costly or costly (c = 0.2). (PDF) [file pcbi.1006179.s003.pdf]

# S4 Figure

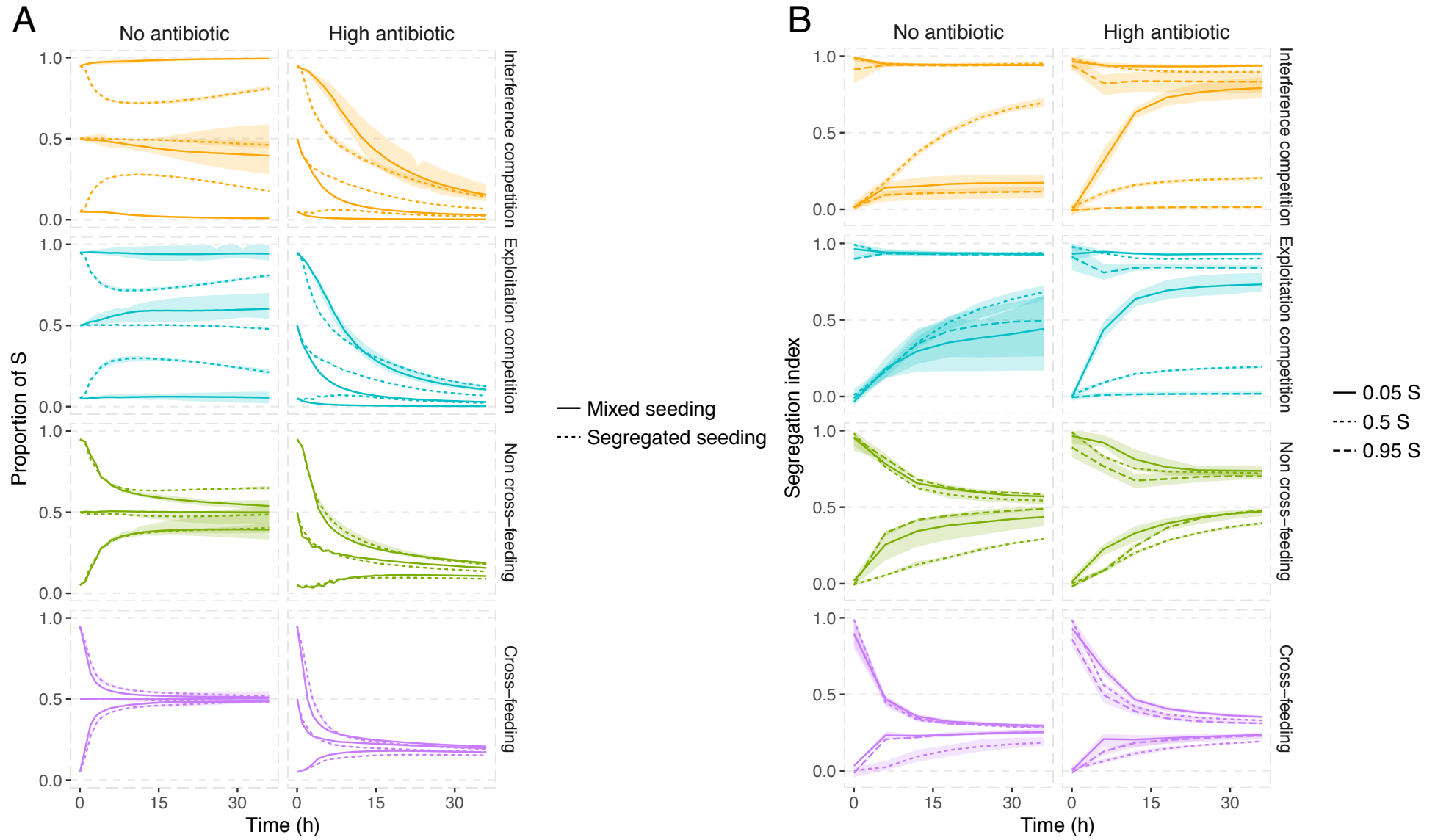

Supplement: S4 Fig — Here we vary both the initial proportion of S cells (5%, 50%, or 95%) and the degree of mixing between S and R cells (mixed, filled line; or segregated, dashed line) at inoculation. A. We can see that, in the interference competition medium, the most common type wins (positive frequency-dependent selection) when no antibiotics are present, but applying antibiotics to the medium shifts the balance towards resistant species being favoured irrespective of initial conditions. In the cross-feeding medium, however, susceptibles merge towards an equilibrium proportion that is independent of initial proportions but that decreases with increasing level of antibiotics. B. Segregation index of communities shown in S4A. See Methods for segregation index description. (PDF) [file pcbi.1006179.s004.pdf]

# S5 Figure

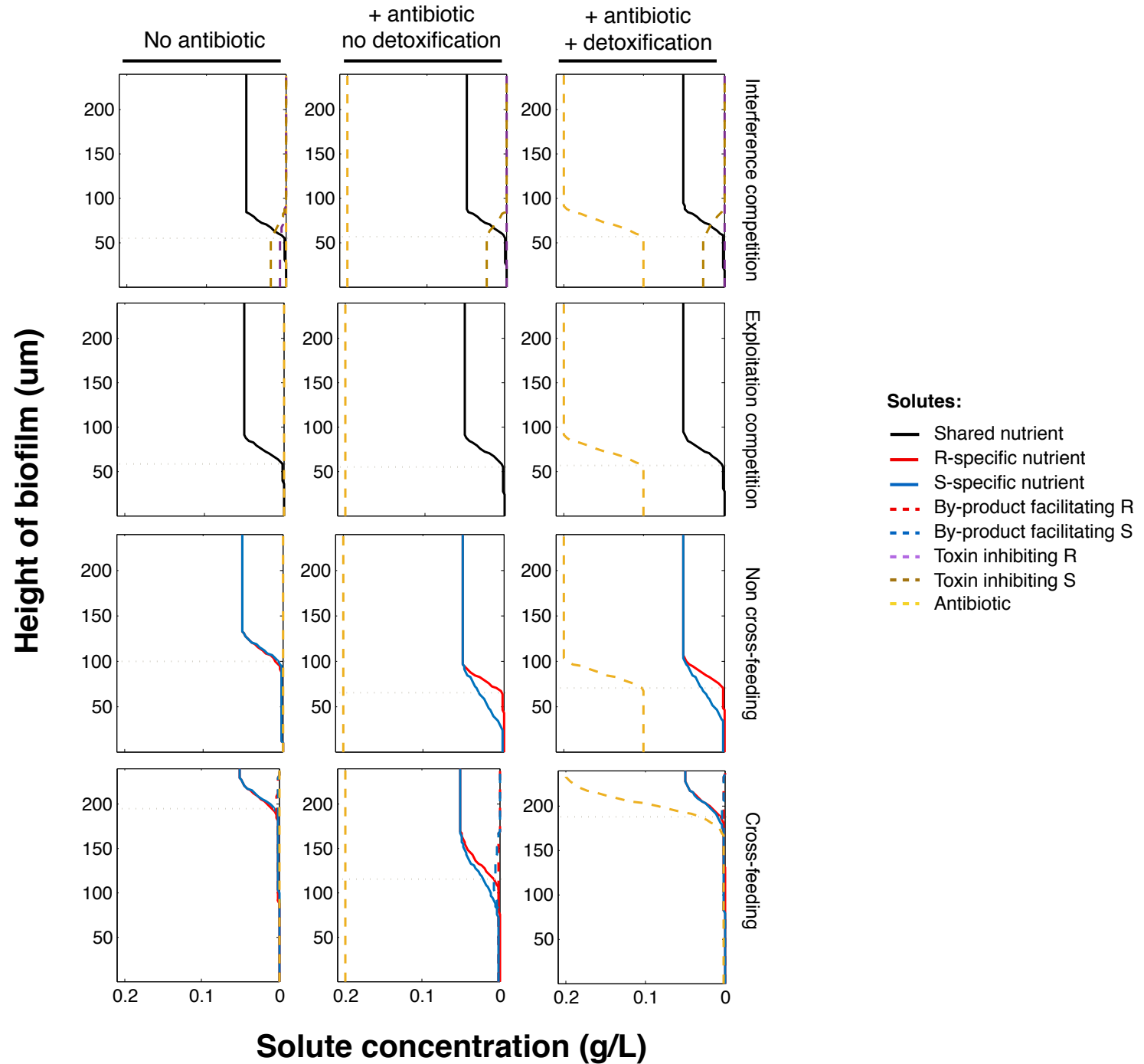

Supplement: S5 Fig — Shown are representative profiles of the average of solute concentrations (nutrients, by-products, toxins, and antibiotic) as a function of community height after 24 hours of growth. The gray horizontal dashed line shows the mean of the height of the biofilm. The two species are seeded randomly and at 1:1. (PDF) [file pcbi.1006179.s005.pdf]

# S6 Figure

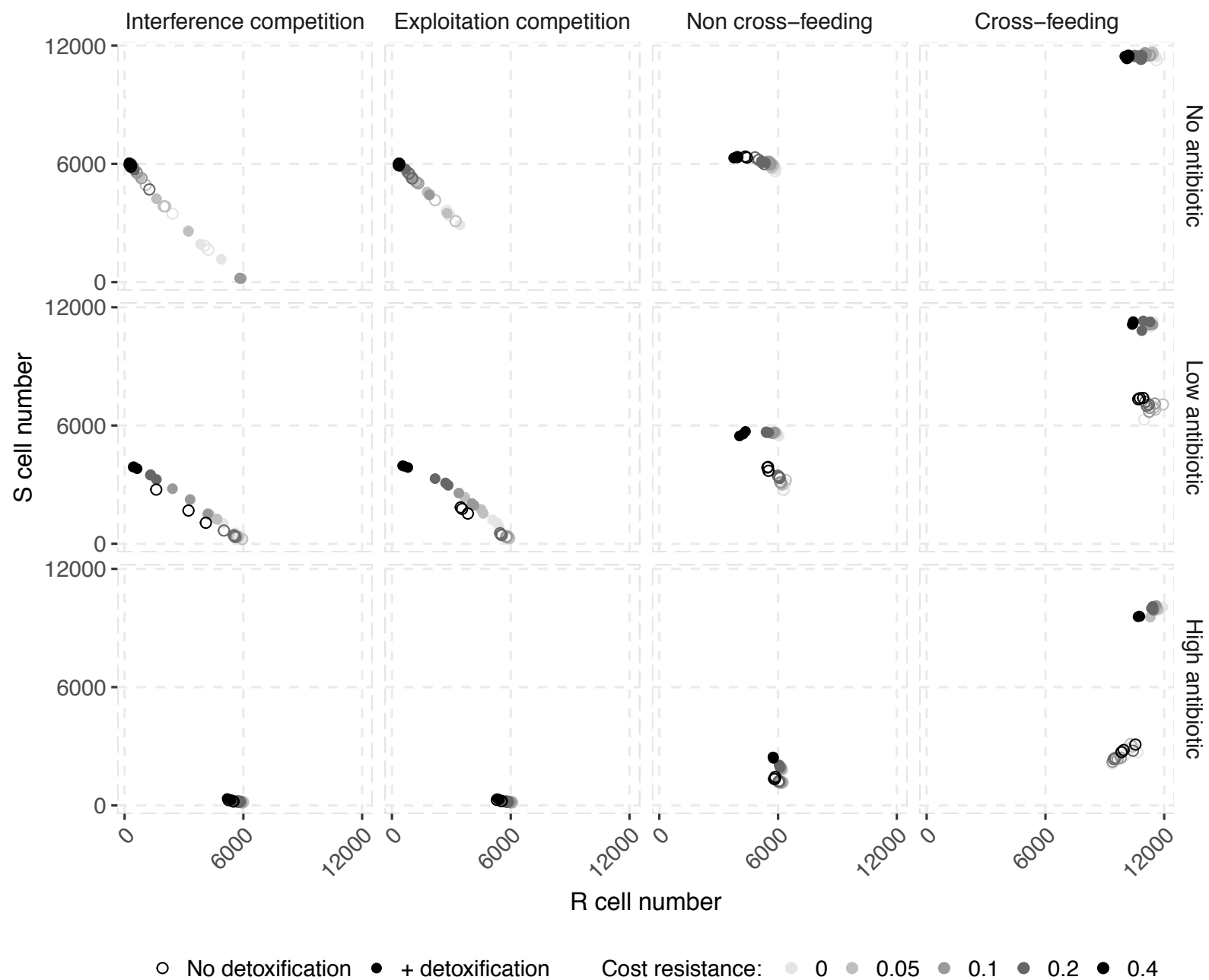

Supplement: S6 Fig — Plotted are the densities after 36h of coculture growth. At inoculation, the two types are seeded randomly and at 1:1. (PDF) [file pcbi.1006179.s006.pdf]

S7 Figure

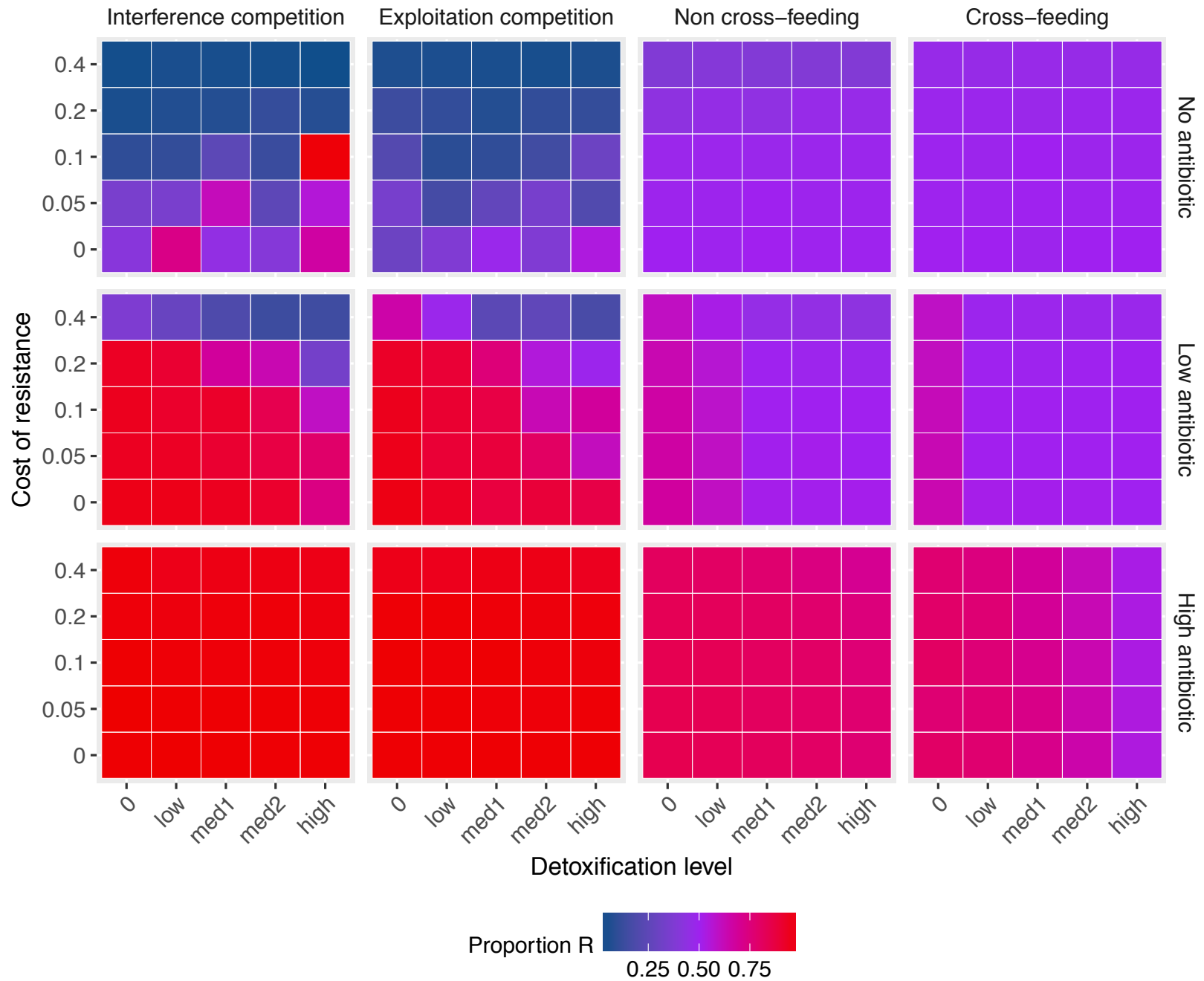

Supplement: S7 Fig — The two types are seeded randomly and at 1:1. (PDF) [file pcbi.1006179.s007.pdf]

# S8 Figure

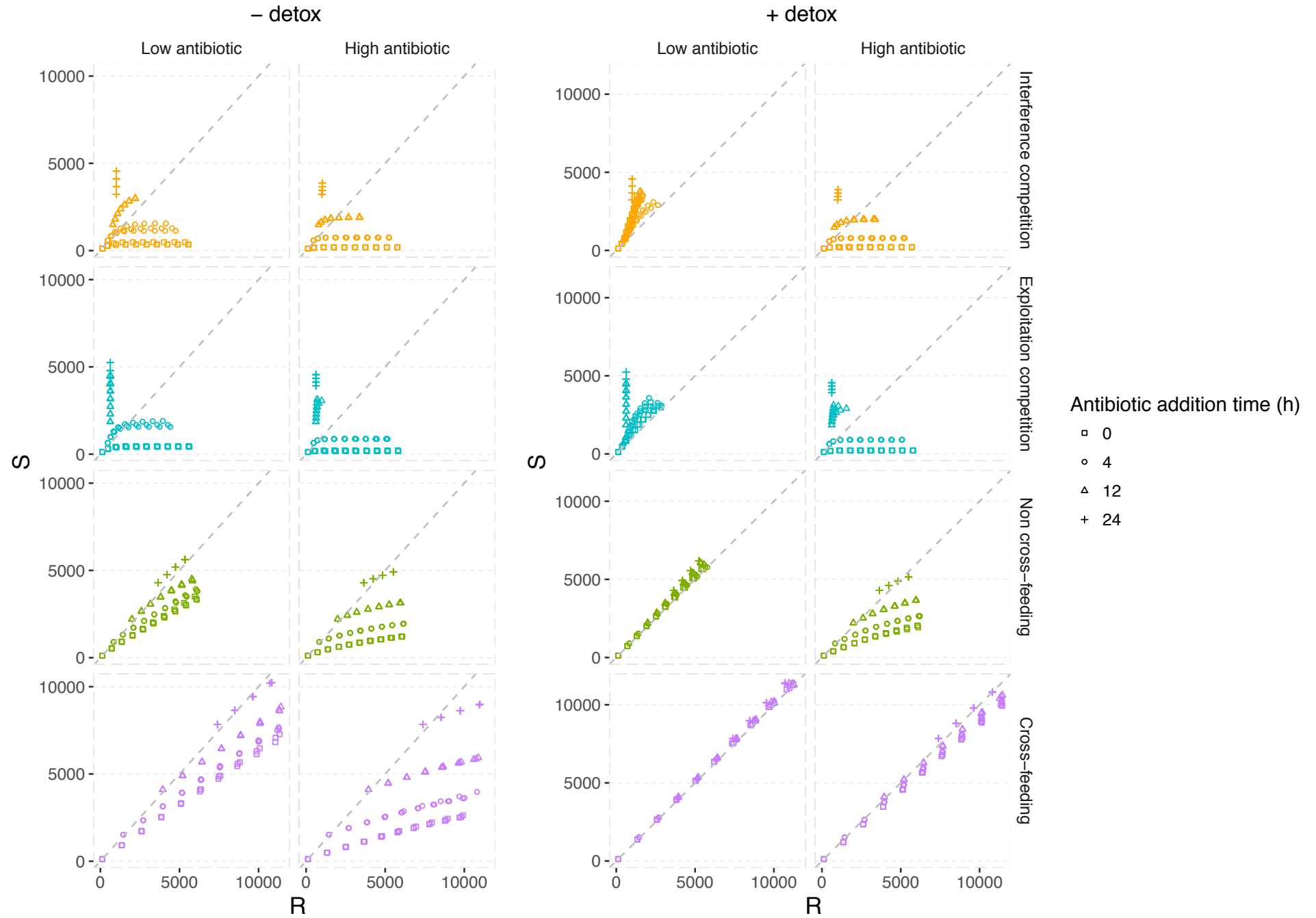

Supplement: S8 Fig — Antibiotics are added at 0h (default), or after 4h, 12h, or 24h of biofilm growth. Shown are the coculture densities of R and S for every 4 hours from the time the antibiotic is added to 36h of biofilm growth in total (ie., growth without antibiotics plus growth with antibiotics). We can see that later antibiotic addition favours susceptibles, and can even prevent the competitive release of the resistant strain. This effect is stronger with detoxification and when antibiotic concentrations are low. In the simulations, resistance is costly (c = 0.2). (PDF) [file pcbi.1006179.s008.pdf]
